# Health economic impact of patients with phenylketonuria (PKU) in France – A nationwide study of health insurance claims data

**DOI:** 10.1016/j.ymgmr.2024.101134

**Published:** 2024-08-20

**Authors:** Jean-Baptiste Arnoux, Claire Douillard, Francois Maillot, Stéphane Bouée, Christian Jacob, Kim Maren Schneider, Julia Theil, Sybil Charrière

**Affiliations:** a Centre de Référence des Maladies Héréditaires du Métabolisme, Hôpital Necker-Enfants Malades, APHP, 149 rue de Sèvres 75015, Paris, France; b Service d'endocrinologie, diabétologie, métabolisme et nutrition, Centre de Référence des Maladies Héréditaires du Métabolisme, Hôpital Huriez, CHU de Lille, 59037 Lille, France; c Service de médecine interne, CHRU et Université de Tours, UMR INSERM 1253 « iBrain », Tours, France; d CEMKA, 43 boulevard du Maréchal Joffre, 92340 Bourg-La-Reine, France; e Xcenda GmbH, Lange Laube 31, 30159 Hannover, Germany; f Hospices Civils de Lyon, Department of endocrinology, diabetologia, metabolic diseases and nutrition, Hôpital Louis Pradel, 69600 BRON, France; g CarMen laboratory, INSERM, INRAE, Université Claude Bernard Lyon 1, 69310 Pierre Bénite, France

**Keywords:** Phenylketonuria (PKU), Healthcare costs, Hospitalizations, Outpatient care, SNDS health insurance data, France

## Abstract

**Background:**

Phenylketonuria (PKU) is an inherited metabolic disease. If left untreated, it can lead to severe irreversible intellectual disability and can cause seizures, behavior disturbance, and white matter disease. This study aimed at evaluating the health economic impact of patients with PKU in France.

**Methods:**

This retrospective observational study used health insurance claims data from the French SNDS (Système National des Données de Santé) database, which contains data from over 66 million French inhabitants. Patients with PKU were identified by ICD-10 diagnosis codes E70.0 (PKU) and E70.1 (Other hyperphenylalaninemia) documented as a chronic condition (affection de longue durée – ALD) or in the inpatient setting in the SNDS database between 2006 and 2018. Patients with PKU were matched to controls without PKU by age, sex, and region. Patients with early- and late-diagnosed PKU were defined as patients born after and before the implementation of nationwide newborn screening in France in 1972, respectively. Outcomes were analyzed for the year 2018.

**Results:**

Overall, 3549 patients with PKU were identified in the database on January 1st, 2018. Of those, 3158 patients versus 15,703 controls with at least one healthcare consumption in 2018 were available for outcome analyses. Patients with PKU had 7.7 times higher healthcare costs than non-PKU controls in 2018 (€11,144 versus 1456 mean costs; *p* < 0.0001). Pharmaceutical costs including dietary amino acid supplements were the cost driver and contributed 80.0% of the overall mean difference (MD) between patients with PKU and matched non-PKU controls. More than half (52.4%) of the mean pharmaceutical costs per patient with PKU was attributable to medical foods including dietary amino acid supplements.

Of the 3158 patients with PKU, 2548 (80.7%) were classified as early-diagnosed and 610 (19.7%) as late-diagnosed. Increased healthcare costs, in comparison to non-PKU controls, were more evident in early-diagnosed patients (€11,263 versus €855 mean costs; 13.2-fold increase; *p* < 0.0001). For patients with late-diagnosed PKU, healthcare costs were 2.7-fold higher compared to matched non-PKU controls (€10,644 versus €3951 mean costs; p < 0.0001). Outpatient pharmaceutical costs accounted for 89.1% of the MD between early-diagnosed patients and controls. Among late-diagnosed patients, 55.5% of the MD were attributable to costs for inpatient care, followed by costs for outpatient care (23.9%) and outpatient pharmaceutical costs (20.6%).

**Conclusion:**

The results indicate that PKU is associated with substantially increased health care costs compared to non-PKU controls in France. The health economic impact was most evident in patients with early-diagnosed PKU due to increased outpatient pharmaceutical costs, especially for medical foods including dietary amino acid supplements. For late-diagnosed and by definition older patients with PKU, the excess costs compared with matched controls were mostly driven by costs for inpatient care.

## Highlights

• Large study on health economic impact of PKU in France with French SNDS database.
• Patients with PKU had 7.7 times higher costs compared with non-PKU controls.
• Impact was most evident in early-diagnosed PKU with drug costs as main driver.
• Costs of patients with late-diagnosed PKU were driven by inpatient care costs.
• Mortality was higher for patients with PKU.

Submission Guidelines: https://www.elsevier.com/journals/molecular-genetics-and-metabolism/1096-7192/guide-for-author

## 1 Introduction

Phenylketonuria (PKU) is a rare inherited metabolic disorder. It is characterized by a deficiency in the enzyme phenylalanine hydroxylase (PAH) which leads to an accumulation of the essential amino acid phenylalanine (Phe) to potentially toxic levels in the blood and brain [1]. Different phenotypes of PAH deficiency from mild hyperphenylalaninemia to classic PKU (severe PAH deficiency) exist depending on residual PAH activity [2]. At the time of this study, patients were classified into classic PKU (>1200 μmol/L), mild PKU (600–1200 μmol/L), and mild hyperphenylalaninemia (120–600 μmol/L) according to untreated plasma Phe [3]. The incidence of PKU varies worldwide. In Europe, approximately 1 in 8000 newborns is diagnosed with PAH deficiency [4] and 1 in 12,500 newborns is diagnosed with PKU [5]. The mean incidence in France from 1972 until 2016 was approximately 1:10,000 newborns for PAH deficiency (including classic PKU, mild PKU and mild hyperphenylalaninemia) and 1:16,500 newborns for classic and mild PKU [6].

If left untreated, PKU results in severe irreversible intellectual disability, growth failure, hypopigmentation, motor deficits, ataxia, and seizures [2,7]. Therefore, early diagnosis is crucial and treatment with a low-Phe diet is recommended for all patients with untreated blood Phe concentrations over 360 μmol/L before the age of 12 years and over 600 μmol/L after the age of 12 years to prevent irreversible impairments in children and minimize morbidity in adolescents and adults [1,7,8]. Adjuvant treatment with sapropterin can benefit a subset of patients with residual PAH activity who show sufficient response to the BH4 (tetrahydrobiopterin) testing protocol [1]. Enzyme substitution therapy with phenylalanine ammonia lyase provides an alternative treatment option to a low-Phe diet for adults but was not available in France at the time of the study [7,9]. Newborn screening (NBS) for PKU facilitates early diagnosis and management of PKU and helps to improve patients' prognosis [10]. In France, a nationwide NBS for PKU was implemented in 1972 [6].

Even though the combination of NBS and available treatment options enables the prevention of most severe manifestations of PKU, patients with PKU were shown to have increased rates of age-related comorbidities like osteoporosis, hypertension, dyslipidemias, diabetes, and ischemic heart disease as well as increased rates of depression and obesity [[11], [12], [13], [14], [15]]. Accordingly, the health economic impact of PKU was shown to be substantial considering not only PKU-specific but all received healthcare [12,16].

The aim of this study was to gain insights into the health economic impact of patients with PKU in France using the French health insurance database SNDS (Système National des Données de Santé).

## 2 Materials and methods

### 2.1 Data source

This retrospective observational study of patients with PKU in France utilized data from the French health insurance claims database SNDS. This nationwide database covers 98.8% of the French population (nearly 66 million people) and represents one of the largest medico-administrative databases in the world.

Apart from administrative information such as year of birth, gender, county of residence, and status as beneficiary of special care, the database includes outpatient and inpatient healthcare resource utilization and associated costs from a third-party payer perspective as well as information about long-term diseases, occupational accidents, occupational disorders, and death. A detailed description of the SNDS database can be found elsewhere [14,15].

All required approvals for data access and analysis have been obtained for this study.

### 2.2 Study population

#### 2.2.1 Patients with PKU

Patients with PKU were identified in the enrollment period between January 1st, 2006 and December 31st, 2018. They were defined as a) patients who benefited from a comprehensive reimbursement due to Affection de Longue Durée (ALD) number 17 registered with an ICD-10 diagnosis codes for PKU (E70.0 Classical phenylketonuria or E70.1 Other hyperphenylalaninaemias) or b) were affiliated to a health insurance scheme and had at least one PKU-specific ICD-10 diagnosis code (E70.0 or E70.1) during a hospitalization (primary or secondary discharge diagnosis). All identified patients with PKU needed to be alive on January 1st, 2018 or born in the year 2018. The overall population of patients with PKU consists of patients born before and after the introduction of the NBS.

Patients born after the implementation of nationwide NBS for PKU in France (1972) were categorized as patients with early-diagnosed PKU. Patients born before the nationwide implementation of NBS for PKU in France in 1972 were categorized as late-diagnosed PKU patients. Even though the NBS was introduced earlier in some regions, the cut-off year 1972 was chosen, because by then the NBS was available nationwide and it was deemed most important to identify a clean population of patients with early-diagnosed PKU. This is considered a conservative approach to estimate healthcare consumption as patients who were diagnosed via NBS but classified as part of the late-diagnosed PKU cohort are in fact early-diagnosed and therefore, presumably have less comorbidities and a reduced pharmaceutical treatment burden. Further limitations of this approach and consequences for classification into early- and late-diagnosed patients with PKU are described in the discussion further below.

#### 2.2.2 Non-PKU controls

To assess excess healthcare resource utilization and costs related to PKU, controls from the general population without PKU were selected at random from the SNDS database.

For each eligible patient with PKU, up to five controls without a recorded PKU diagnosis during the enrolment period were drawn from the database via a direct, exact matching without replacement on age, sex, and region.

The status of the matching parameters was determined on January 1st, 2018. Like the patients with PKU, the controls needed to be alive on January 1st, 2018 or newborns in the year 2018.

### 2.3 Outcomes

The prevalence of PKU was calculated for December 31st, 2018. The mortality was analyzed for the entire year 2018.

The demographic characteristics age and gender were analyzed on January 1st, 2018. Outpatient and inpatient healthcare resource utilization as well as healthcare costs were analyzed for the entire year 2018. Healthcare costs were defined from the third-party payer perspective. They reflect the costs reimbursed by the national health insurance in France.

### 2.4 Statistical methods

Continuous variables were described in terms of mean, standard deviation, minimum, quantile 25, median, quantile 75, and maximum. Differences between patients with PKU and matched controls were tested using a Student's *t*-test or analysis of variance when distribution was close to normal (non-significant Shapiro-Wilk test). If this was not the case, non-parametric tests were used (Wilcoxon, Kruskal-Wallis).

Categorical variables were described in terms of counts and percentages. Differences between patients with PKU and matched controls were tested using the Chi^2^ test. In case the theoretical sample sizes were <5, Yates continuity correction or Fisher's exact test was used.

Mortality was compared between patients with PKU and matched controls using the Kaplan-Meier method and log-rank test. All tests were interpreted two-tailed with an alpha of 5%.

## 3 Results

### 3.1 Study population

**Fig. 1:**
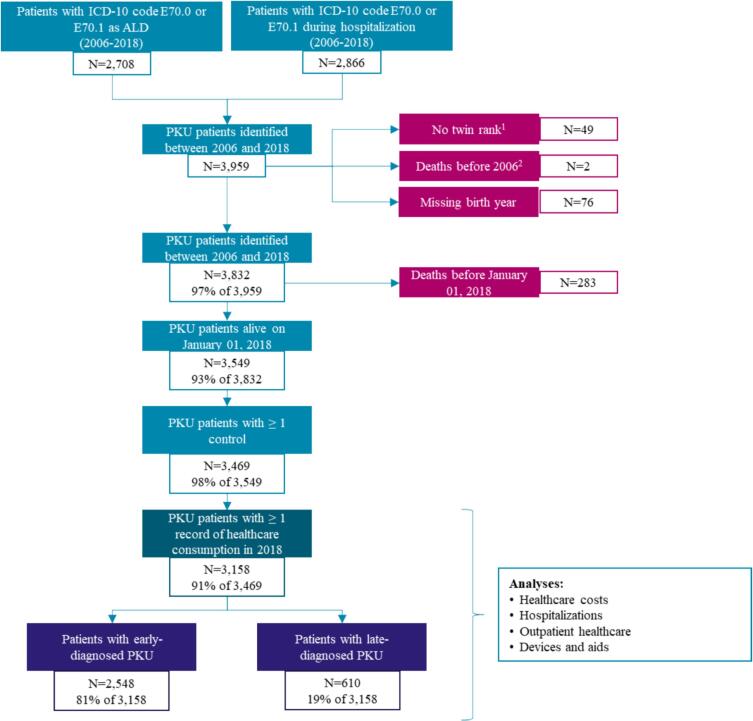
Flowchart of patient selection. ALD, Affection de Longue Durée; PKU, phenylketonuria. ^1^For twins, there is usually a rank that allows the differentiation of twins in the database. The rank was unavailable for 49 individuals. ^2^The transferred data included a PKU diagnosis between 2006 and 2018 for two patients who had already died before 2006. This is a data error, and the two patients were excluded accordingly.

Overall, 3549 patients with PKU who were alive on January 1st, 2018, were identified in the database. Of those, 3469 patients could be matched to 17,170 controls without PKU (96.6% of patients with PKU were matched to 5 controls, 2.5% to 4 controls, 0.4% to 3 controls, 0.2% to 2 controls, and 0.3% to 1 control). For the outcome analyses, it was required that patients and matched control(s) had at least one documented healthcare consumption in 2018. This reduced the number of patients with PKU to 3158 and the number of matched controls to 15,703 (Fig. 1).

Among the 3158 patients with PKU, 2548 (80.7%) were classified as early-diagnosed and 610 (19.7%) as late-diagnosed. They were matched to 12,656 and 3047 controls, respectively (Fig. 1).

Mean age of the 3158 patients with PKU in 2018 was 27.9 years (standard deviation 23.6 years). The sex distribution was balanced with 46.7% female and 53.3% male patients. Patients with early-diagnosed PKU were on average 18.2 years old (standard deviation 12.5 years) and patients with late-diagnosed PKU 68.5 years old (standard deviation 13.5 years). Women and men were evenly distributed among early and late-diagnosed patients. Details on the age, sex, and region distribution can be found in Table 1.

**Table 1:** Sociodemographic data of patients with PKU and non-PKU controls.

	**Overall**		**Early-diagnosed**		**Late-diagnosed**	
	**Patients with PKU**	**Non-PKU controls**	**Patients with PKU**	**Non-PKU controls**	**Patients with PKU**	**Non-PKU controls**
N	3158	15,703	2548	12,656	610	3047
Sex^a^						
Male	1475 (46.7%)	7332 (46.7%)	1163 (45.6%)	5775 (45.6%)	312 (51.1%)	1557 (51.1%)
Female	1683 (53.3%)	8370 (53.3%)	1385 (54.4%)	6880 (54.4%)	298 (48.9%)	1490 (48.9%)
Age in 2018						
Mean (standard deviation)	27.9 (23.6)	27.9 (23.6)	18.2 (12.5)	18.2 (12.5)	68.5 (13.5)	68.5 (13.5)
Median / Min / Max	21.0 / 0.0 / 98.0	21.0 / 0.0 / 98.0	16.0 / 0.0 / 46.0	16.0 / 0.0 / 46.0	69.0 / 47.0 / 98.0	69.0 / 47.0 / 98.0
Regions						
Not available	13	80	12	66	1	14
Grand-Est	400 (12.7%)	1984 (12.7%)	322 (12.7%)	1602 (12.7%)	78 (12.8%)	382 (12.6%)
Nouvelle-Aquitaine	197 (6.3%)	936 (6.0%)	178 (7.0%)	848 (6.7%)	19 (3.1%)	88 (2.9%)
Auvergne-Rhône-Alpes	353 (11.2%)	1786 (11.4%)	300 (11.8%)	1492 (11.9%)	53 (8.7%)	294 (9.7%)
Bourgogne-Franche-Comté	175 (5.6%)	813 (5.2%)	135 (5.3%)	612 (4.9%)	40 (6.6%)	201 (6.6%)
Bretagne	107 (3.4%)	535 (3.4%)	68 (2.7%)	344 (2.7%)	39 (6.4%)	191 (6.3%)
Centre-Val-de-Loire	111 (3.5%)	535 (3.4%)	86 (3.4%)	412 (3.3%)	25 (4.1%)	123 (4.1%)
Ile de France	496 (15.8%)	2551 (16.3%)	414 (16.3%)	2149 (17.1%)	82 (13.5%)	402 (13.3%)
Occitanie	235 (7.5%)	1099 (7.0%)	206 (8.1%)	961 (7.6%)	29 (4.8%)	138 (4.5%)
Hauts-de-France	392 (12.5%)	2044 (13.1%)	263 (10.4%)	1379 (11.0%)	129 (21.2%)	665 (21.9%)
Normandie	170 (5.4%)	795 (5.1%)	144 (5.7%)	693 (5.5%)	26 (4.3%)	102 (3.4%)
Pays de la Loire	163 (5.2%)	795 (5.1%)	128 (5.0%)	616 (4.9%)	35 (5.7%)	179 (5.9%)
Provence-Alpes-Côte d'Azur + Corse	325 (10.3%)	1608 (10.3%)	275 (10.8%)	1361 (10.8%)	50 (8.2%)	247 (8.1%)
Territoire d'outre-mer	21 (0.7%)	142 (0.9%)	17 (0.7%)	121 (1.0%)	4 (0.7%)	21 (0.7%)

a Sex was not available for one control from the early-diagnosed group.

### 3.2 Prevalence and mortality

Of the 3549 PKU patients who were alive on January 1st, 2018, 3504 patients survived until December 31st, 2018. This corresponds to a point prevalence of 5.2 per 100,000 inhabitants in France on December 31st, 2018 considering a French population of 67.0 million inhabitants on January 1st, 2019 [17].

**Fig. 2:**
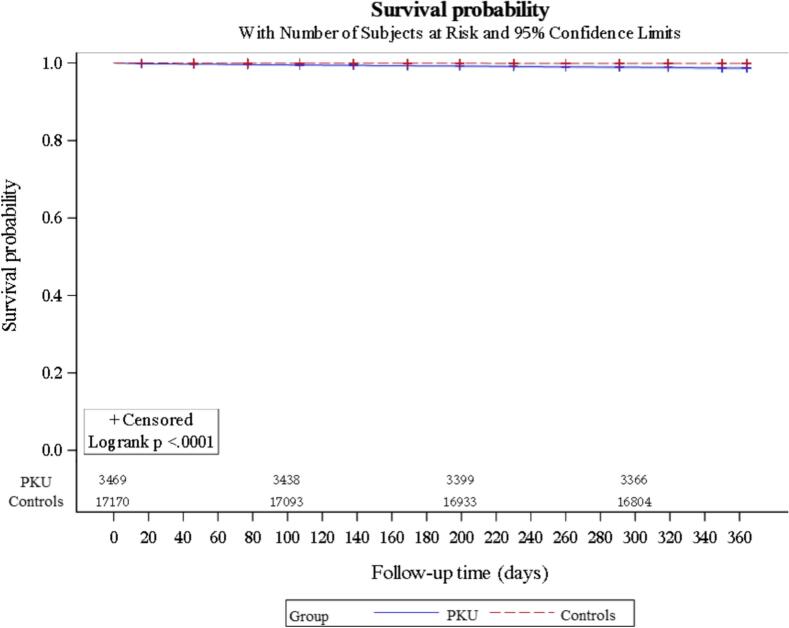
Kaplan-Meier plot of survival probability in 2018 of patients with PKU and matched controls.

Of the 3469 patients with PKU who could be matched to 17,170 controls, 44 patients with PKU (1.3%) and 19 controls without PKU (0.1%) died in the year 2018 (*p* < 0.0001). The Kaplan-Meier plot is shown in Fig. 2.

### 3.3 All patients with PKU

#### 3.3.1 Healthcare costs

**Fig. 3:**
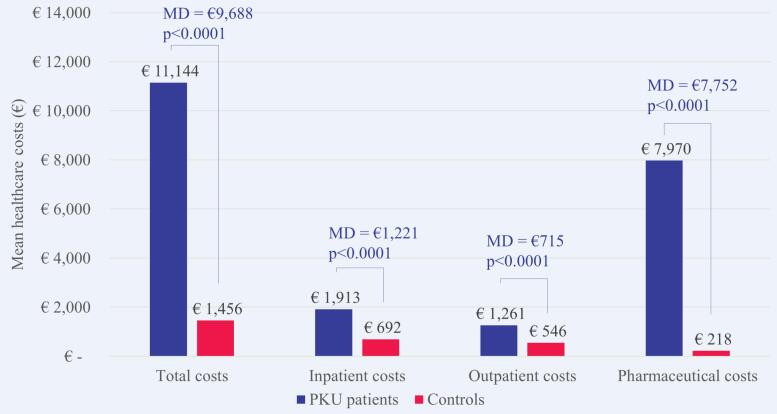
Mean healthcare costs in 2018 per patient with PKU versus matched control. MD, mean difference; PKU, phenylketonuria.

The mean healthcare costs in 2018 were €11,144 per patient with PKU and €1456 per matched control. This means that a patient with PKU on average had 7.7 times higher costs than a matched control without PKU. The mean difference (MD) was €9688 (*p* < 0.0001). The healthcare costs were higher for patients with PKU than matched controls in each cost domain as shown in Fig. 3, especially the costs for outpatient pharmaceutical treatment (MD €7752; p < 0.0001). They accounted for 80.0% of the overall MD, followed by inpatients costs (12.6%) and outpatient costs (7.4%).

**Fig. 4:**
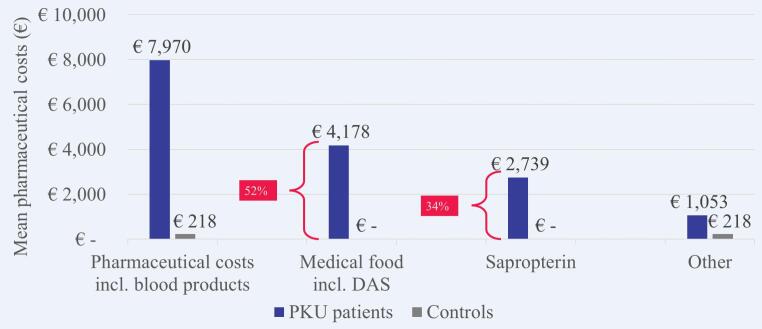
Mean costs for specific pharmaceuticals in 2018 per patient with PKU versus matched control. DAS, dietary amino acid supplements; PKU, phenylketonuria.

More than half (52.4%) of the mean pharmaceutical costs per patient with PKU were attributable to medical foods including dietary amino acid supplements and 34.4% to sapropterin (Fig. 4), although medical foods were prescribed to only 1244 (39.4%) and sapropterin to only 362 (11.5%) patients with PKU.

#### 3.3.2 Hospitalizations and outpatient healthcare

In 2018, 29.1% of patients with PKU were hospitalized, including one-day hospitalizations as well hospitalizations with overnight stay, compared with 16.1% of matched controls (*p* < 0.0001). Hospitalized patients with PKU tended to have a longer mean length of stay in terms of nights spent at the hospital (5.6 nights) than hospitalized controls (4.2 nights), but the difference was not statistically significant (*p* = 0.138). However, hospitalized patients with PKU on average presented with more hospital stays than hospitalized controls (2.7 versus 1.7 mean hospital stays; *p* < 0.0001).

Considering only one-day hospitalizations without an overnight stay, these were also elevated in patients with PKU (17.1% versus 7.6%; p < 0.0001).

Regarding outpatient healthcare, the proportion of patients with PKU and matched controls who consulted a general practitioner was similar (83.8% versus 84.4%; *p* = 0.38). Among those, patients with PKU visited their general practitioner more frequently than controls (5.3 versus 4.4 mean visits; *p* < 0.0001). Consultations of specialists were comparable between patients with PKU and matched controls (61.0% versus 62.7%; *p* = 0.0581; 4.0 versus 3.4 mean visits; *p* = 0.1717). More patients with PKU than matched controls had outpatient consultations in the hospital (62.6% versus 27.6%; *p* < 0.0001). Among those, the mean number of consultations was higher for patients with PKU than controls (3.8 versus 3.2 consultations; *p* < 0.0001). Paramedical care such as consultations of nurses or physiotherapists was received by more patients with PKU than matched controls. Nurses were consulted by 46.8% of patients with PKU and 25.3% of matched controls (*p* < 0.0001). Furthermore, 15.8% of patients were treated by a physiotherapist compared with 12.6% of controls (p < 0.0001).

Medical devices were prescribed to more patients with PKU than controls (55.7% versus 46.6%; p < 0.0001) and among those, the mean number of prescribed devices was higher per patient with PKU (7.1 versus 4.2 mean devices; p < 0.0001). In accordance, more patients with PKU than matched controls required transportation for medical reasons (11.0% versus 3.9%; p < 0.0001) and among those, the mean number of transports was higher per patient with PKU (10.8 versus 7.7 mean transports; p < 0.0001).

### 3.4 Patients with early-diagnosed PKU

#### 3.4.1 Healthcare costs

**Fig. 5:**
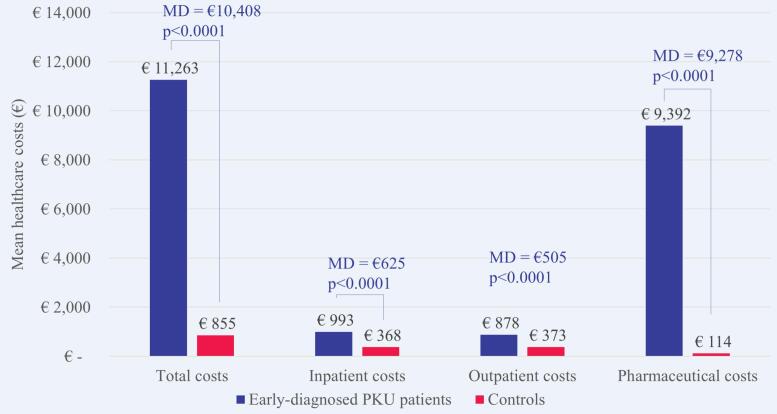
Mean healthcare costs in 2018 per patient with early-diagnosed PKU versus matched control. MD, mean difference; PKU, phenylketonuria.

**Fig. 6:**
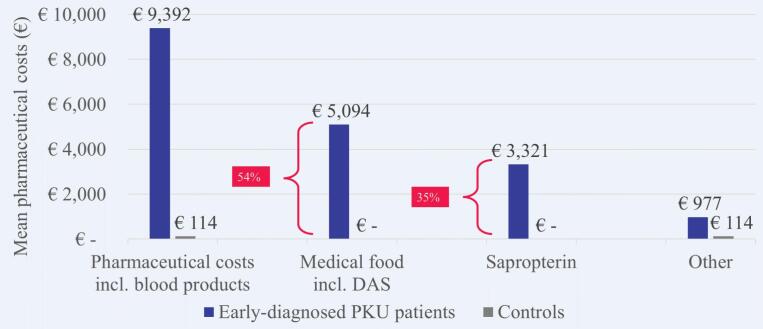
Mean costs for specific pharmaceuticals in 2018 per patient with early-diagnosed PKU versus matched control. DAS, dietary amino acid supplements; PKU, phenylketonuria.

The mean healthcare costs in 2018 were €11,263 per patient with early-diagnosed PKU and €855 per matched control without PKU. The MD was €10,408 (p < 0.0001) and patients with early-diagnosed PKU caused on average 13.2 times higher costs than matched controls. Higher mean healthcare costs were observed in each cost domain and most evident for outpatient pharmaceutical costs with a MD of €9278 (Fig. 5). Costs for outpatient pharmaceutical treatment accounted for 89.1% of the overall MD, followed by inpatient costs (6.0%) and outpatient costs (4.9%). Medical foods including dietary amino acid supplements attributed 54.2% and sapropterin 35.4% of the pharmaceutical costs for patients with early-diagnosed PKU (Fig. 6), although medical were prescribed to only 1223 (48.0%) and sapropterin to only 358 (14.1%) patients with early-diagnosed PKU.

To gain additional insights into the health econocmic burden of PKU in children an additional analysis was performed, which stratified the overall PKU cohort in patients aged <16 years and patients aged ≥16 years. Compared to their matched controls without PKU, patients aged <16 years had 19.6-fold increased healthcare costs (€12,993 versus €664; p 〈00001) and patients aged ≥16 years had 5.0-fold increased healthcare costs (€9937 versus 1972; *p* < 0.0001). The cost increase in patients <16 years old (mean age 7.5 years) was more pronounced than in the group of early-diagnosed patients (mean age 18.2 years).

#### 3.4.2 Hospitalizations and outpatient healthcare

More patients with early-diagnosed PKU than matched controls were hospitalized in 2018, including one-day hospitalizations as well as hospitalizations with overnight stay (25.5% versus 13.7%; *p* < 0.0001). Hospitalized patients with early-diagnosed PKU on average presented with a higher number of hospital stays than hospitalized controls (1.9 versus 1.4 mean hospital stays; *p* < 0.0001). The mean length of stay in terms of nights spent at the hospital was 3.2 nights for both hospitalized patients with early-diagnosed PKU and controls, while the median length of stay showed that hospitalized controls remained in hospital longer than patients with early-diagnosed PKU (2 versus 0 median nights; *p* < 0.0001).

Considering only one-day hospitalizations without an overnight stay, these were also elevated in patients with early-diagnosed PKU (16.8% versus 6.1%; p < 0.0001).

Concerning outpatient healthcare, the proportion of patients with early-diagnosed PKU and matched controls who consulted a general practitioner was comparable (82.8% versus 83.0%; *p* = 0.7974). Among those, patients with early-diagnosed PKU visited their general practitioner more frequently than controls (4.6 versus 3.9 mean visits; *p* < 0.0001). Consultations of specialists were also similar between patients with early-diagnosed PKU and matched controls (57.9% versus 59.1%; *p* = 0.2748; 3.2 versus 3.1 mean visits; *p* = 0.8747). More patients with early-diagnosed PKU than matched controls had outpatient consultations in the hospital (65.0% versus 26.0%; *p* < 0.0001). Among those, the average number of consultations was higher for patients than controls (3.2 versus 2.9 mean consultations; *p* < 0.0001). Paramedical care including consultations of nurses or physiotherapists was received by more patients with early-diagnosed PKU than matched controls. Nurses were consulted by 41.8% of patients with early-diagnosed PKU and 17.6% of matched controls (*p* < 0.0001), and physiotherapists treated 11.4% of patients with early-diagnosed PKU versus 9.6% of matched controls (*p* = 0.006).

Medical devices were prescribed to more patients with early-diagnosed PKU than matched controls (51.7% versus 42.6%; p < 0.0001) and among those, the average number of prescribed devices was higher per patient than control (4.4 versus 3.1 mean devices; p < 0.0001). Transportation for medical reasons were needed by more patients with early-diagnosed PKU than matched controls (6.7% versus 1.9%; p < 0.0001). Among those, the mean number of transports was higher per patient with early-diagnosed PKU than per control (7.7 versus 6.7 transports; p < 0.0001).

### 3.5 Patients with late-diagnosed PKU

#### 3.5.1 Healthcare costs

**Fig. 7:**
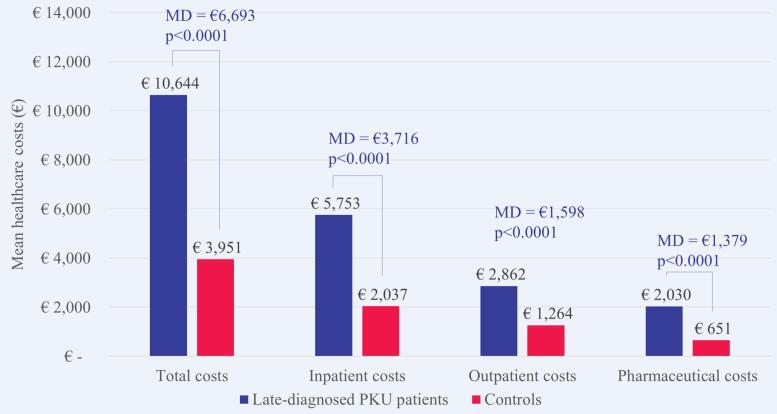
Mean healthcare costs in 2018 per patient with late-diagnosed PKU versus matched control. MD, mean difference; PKU, phenylketonuria.

The mean healthcare costs in 2018 were €10,644 per patient with late-diagnosed PKU and €3951 per matched control without PKU. Consequently, a patient with late-diagnosed PKU on average had 2.7 times higher costs than a matched control. The MD was €6693 (*p* < 0.0001). Higher healthcare costs for patients with late-diagnosed PKU than matched controls were evident in all cost domains, especially for costs for inpatient care (Fig. 7). Inpatient costs accounted for 55.5% of the overall MD, followed by outpatient costs (23.9%) and outpatient pharmaceutical costs (20.6%).

**Fig. 8:**
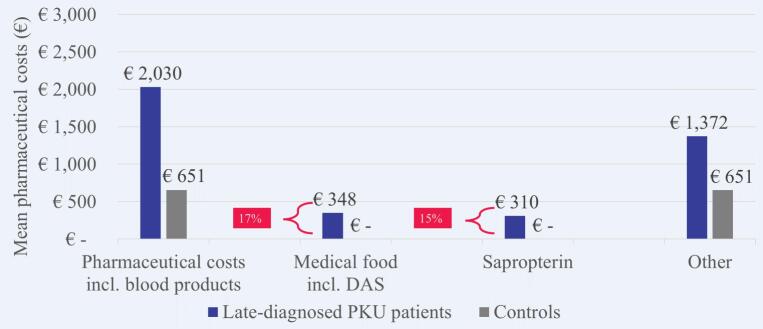
Mean costs for specific pharmaceuticals in 2018 per patient with late-diagnosed PKU versus matched control. DAS, dietary amino acid supplements; PKU, phenylketonuria.

Nearly one fifth (17.2%) of the mean pharmaceutical costs per patient with late-diagnosed PKU were attributable to medical foods including dietary amino acid supplements and 15.2% to sapropterin (Fig. 8). Medical foods were prescribed only to 21 (3.4%) patients with late-diagnosed PKU and four (0.7%) patients were treated with sapropterin.

#### 3.5.2 Hospitalizations and outpatient healthcare

In 2018, 43.9% of patients with late-diagnosed PKU were hospitalized including one-day hospitalizations as well as hospitalizations with overnight stay, compared with 25.9% of matched controls (*p* < 0.0001). Hospitalized patients with late-diagnosed PKU on average had a longer length of stay in terms of nights spent at the hospital than hospitalized controls (11.5 versus 6.6 mean nights; *p* < 0.0001) and were hospitalized more frequently (4.5 versus 2.3 mean hospital stays; p < 0.0001).

Considering only one-day hospitalizations without an overnight stay, these were also elevated in patients with late-diagnosed PKU (18.7% versus 13.5%; *p* = 0.0009).

Regarding outpatient healthcare, an equal proportion of patients with late-diagnosed PKU and matched controls consulted a general practitioner (87.9% versus 90.2%; *p* = 0.0842). Among those, patients with late-diagnosed PKU visited their general practitioner more frequently than controls (8.4 versus 6.4 mean visits; *p* < 0.0001). The proportion of patients with late-diagnosed PKU who consulted a specialist was lower compared with matched controls (73.6% versus 77.9%; *p* < 0.0207), but among those with at least one consultation of a specialist, the average number of visits was higher for patients than controls (6.6 versus 4.6 mean visits; *p* < 0.0001). Outpatient consultations in the hospital were more frequent for patients with late-diagnosed PKU than matched controls (52.6% versus 34.4%; *p* < 0.0001). Among those, the mean number of consultations was higher for patients than controls (6.5 versus 4.2 consultations; *p* = 0.0048). Paramedical care like consultations of nurses or physiotherapists was received by more patients with late-diagnosed PKU than matched controls. Nurses were consulted by 67.7% of patients with late-diagnosed PKU and 57.6% of matched controls (p < 0.0001). Physiotherapists treated 34.4% of the patients with late-diagnosed PKU and 24.9% of matched controls (p < 0.0001).

Medical devices were prescribed to more patients with late-diagnosed PKU than matched controls (72.1% versus 63.2%; p < 0.0001) and among those, the number of prescribed devices was higher per patient with late-diagnosed PKU than per control (15.0 versus 7.3 mean devices; p < 0.0001). Transportation for medical reasons was needed by more patients with late-diagnosed PKU than matched controls (28.9% versus 11.9%; p < 0.0001) and among those, the mean number of transports was higher per patient (13.8 versus 8.4 mean transports; *p* = 0.0078).

## 4 Discussion

This large study of PKU patients in France analyzed the French health insurance claims database SNDS covering >98% of the French population. The results indicate that PKU has a substantial health economic impact in France. Compared with an age-, sex-, and region-matched control group without PKU from the general French population, patients with PKU had 7.7 times higher mean healthcare costs than controls in 2018. Costs for outpatient pharmaceutical treatment were the main cost driver and contributed 80% of the overall mean cost difference between patients with PKU and matched controls. More than half of the pharmaceutical costs (52%) incurred by patients with PKU was attributable to medical foods including dietary amino acid supplements, although only 39% of patients were treated with them.

The increased health economic impact was most evident in patients with early-diagnosed PKU. They had 13.2 times higher healthcare costs in 2018 than matched controls, especially due to outpatient pharmaceutical costs that contributed 89% of the overall mean cost difference between patients and controls. Again, more than half of the pharmaceutical costs (54%) were attributable to medical foods including dietary amino acid supplements, but only 48% of patients with early-diagnosed PKU received them. Sapropterin contributed 35% of the pharmaceutical costs, meaning that PKU-specific treatments explained nearly 90% of the pharmaceutical costs of patients with early-diagnosed PKU. Patients with late-diagnosed PKU had 2.7 times higher healthcare costs than matched controls in 2018. As opposed to early-diagnosed patients, the main cost driver for late-diagnosed patients were costs for inpatient care which contributed 56% of the overall mean cost difference. Another notable finding is that only 3% of patients with late-diagnosed PKU received medical foods including dietary amino acid supplements. This might be due to changes in the French recommendations of 2010, which previously allowed an enlarged diet for adult patients as long as levels of Phe remained <1200 μmol/L and were reinforced to recommend strict diet for life only in 2018. Moreover, depending on the neurological state and the severity of further PKU-related sequelae, the presciption of dietary amino acid supplements can be difficult to consider for physicians or patients may have difficulties to be compliant with their diet.

Higher healthcare costs for younger patients with PKU were also shown in an additional analysis that stratified the overall PKU cohort in patients aged <16 years and patients aged ≥16 years. Compared to their matched controls without PKU, patients aged <16 years had 19.6-fold increased healthcare costs and patients aged ≥16 years had 5.0-fold increased healthcare costs. The cost increase in patients <16 years old (mean age 7.5 years), which was more pronounced than in the group of early-diagnosed patients (mean age 18.2 years), demonstrates that the health economic impact of PKU is especially high in children with PKU.

In accordance with these findings, the study showed increased hospitalization and outpatient healthcare rates for patients with PKU compared with matched controls in 2018. More patients than controls were hospitalized in 2018, especially among patients with late-diagnosed PKU. The increase in one-day hospitalizations could be attributed to more consultations with dieticians or bone density measurements for patients with PKU. Additionally, outpatient consultations in the hospital and paramedical care including consultations of nurses or physiotherapists were more frequent in patients with PKU, irrespective if early- or late-diagnosed, compared with matched controls without PKU. Furthermore, medical devices and transportations for medical reasons were required for more patients with PKU than controls.

These findings are in line with a study investigating the health economic impact of adult patients with PKU in Germany [16]. The authors found 2.3 times higher annual healthcare costs for adult patients with PKU than age- and sex-matched controls without PKU. In accordance with our study, pharmaceutical costs were the main cost driver in the overall and early-diagnosed PKU cohort and inpatient costs were driving the costs in patients with late-diagnosed PKU. Additionally, as in our study, the health economic impact was particularly evident in adult patients with early-diagnosed PKU. Increased rates of hospitalizations for patients with PKU were also observed in accordance with our study. Overall, the findings from the German study [16] are consistent with our results, however, the mean cost differences between adult patients with PKU in Germany and matched controls were less pronounced than in our study. A U.S. study of adult patients with PKU also found 3.2 times higher healthcare costs for patients with PKU than matched controls [12].

An exploratory study in ten European specialist PKU centers [18] demonstrated that dietary amino acid supplements were the most expensive item in the dietary management of PKU. Our findings also showed that the medical foods including dietary amino acid supplements accounted for more than half of the outpatient pharmaceutical costs in patients with early-diagnosed PKU even though only a fraction of adult patients with early-diagnosed PKU received medical foods including dietary amino acid supplements.

Concerning healthcare costs, it needs to be pointed out that the excess costs shown in our study were expected due to the comparison of individuals with a severe disease and those without the disease. Showing the health economic burden related to the management of a severe genetic disease was the aim of this study. However, this study did not assess the cost-savings associated with the treatment of patients with PKU. Exploring cost-savings would be an important part of understanding and improving the management and treatment of patients with PKU and should be targeted in future research.

The prevalence of PKU on December 31st, 2018 was estimated at 5.2 per 100,000 inhabitants in France in our study. In view of a reported mean incidence in France of 1:10,000 (10:100,000) newborns for PAH deficiency and 1:16,500 (6:100,000) newborns for PKU from 1972 until 2016 [6], the estimation from our study is at the lower bound of reported ranges, even when considering the estimated mortality rate of 1.3% from our study. An explanation may be the possible underrepresentation of older patients with PKU in our study that is described in the following.

This study has some limitations. As mentioned above, older patients with PKU in France seem to be underrepresented in our study as outlined in [14,15]. A possible reason could be that patients who are treated in nursing homes or specialized centers cannot be identified in the SNDS database as their ICD-10 codes are not documented in the SNDS database. Additionally, patients born before the implementation of nationwide NBS, and by definition older patients, may not have been diagnosed at all.

Patients with PKU were identified via the ICD-10 codes E70.0 and E70.1 which may have led to the inclusion of individuals without PKU, because the latter code includes patients with disorders of the BH4 metabolism i.e., non-phenylketonuric hyperphenylalaninemia. As this is a very rare condition [19], the impact of the inclusion of such patients is considered to be small.

Furthermore, the classification of patients with early- and late-diagnosed PKU was based on the year of birth in relation to the nationwide implementation of the NBS in France in 1972. This approach does not account for patients who were born in French regions where the NBS was introduced earlier, for patients who may have been born in other countries without NBS program, or for patients born before NBS but with older siblings with diagnosed PKU (who were therefore diagnosed at birth and became early-diagnosed patients).

Finally, the SNDS database does not provide information about the level of education, income and socio-professional category of the insured. Additionally, as all administrative claims databases, the SNDS database only contains information about care that was actually reimbursed, and neither self-treatment nor the use of prescribed medications that are not reimbursed can be measured. Likewise, it is not possible to evaluate the compliance of patients, since, for example, pharmaceuticals that are prescribed but not filled by the patients are not documented in the SNDS. Moreover, the SNDS database does not contain any clinical information such as laboratory test or medical imaging results.

## 5 Conclusion

This retrospective observational study using the French health insurance claims database SNDS demonstrated that PKU is associated with a high health economic burden in France. Considering all healthcare cost domains, patients with PKU created 7.7 times higher mean healthcare costs in 2018 than age-, sex-, and region-matched controls without PKU. The health economic impact was most evident in patients with early-diagnosed PKU who had 13.2 times higher costs than matched controls.

Costs for outpatient pharmaceutical treatment were revealed to be the main cost driver, with more than half of the pharmaceutical costs being attributable to medical foods including dietary amino acid supplements. For late-diagnosed and by definition older patients with PKU, the excess costs compared with matched controls were mostly driven by costs for inpatient care. The excess costs would likely be even higher if treatment compliance and follow-up of elderly patients improved. The health economic impact of PKU is reflected by increased hospitalization and outpatient healthcare rates of patients with PKU. Indirect costs for the society such as lack of employment or disability allowances, which could not be covered within this study in total, might further contribute to the increased health economic impact.

Future research should investigate if the increased healthcare costs of patients with early-diagnosed PKU will lead to a reduced burden of disease when these patients reach an advanced age.

## Funding

This work was supported by BioMarin Europe Ltd.

## Financial disclosure

JBA, FM, and SC received expert honoraria from BioMarin.

SB is a full-time employee of CEMKA acting as a contractor of Xcenda GmbH for the execution of this study.

CD has received consulting fees from BioMarin and Sanofi Genzyme.

CJ, KMS, and JT are full-time employees of Xcenda GmbH acting as a contractor for BioMarin Europe Ltd. during the conduct of the study and writing of the manuscript.

## CRediT authorship contribution statement

**Jean-Baptiste Arnoux:** Writing – review & editing, Validation, Methodology. **Claire Douillard:** Writing – review & editing, Validation, Methodology. **Francois Maillot:** Writing – review & editing, Validation, Methodology. **Stéphane Bouée:** Writing – review & editing, Validation, Methodology, Formal analysis. **Christian Jacob:** Writing – review & editing, Writing – original draft, Validation, Methodology. **Kim Maren Schneider:** Writing – review & editing, Writing – original draft, Validation, Methodology. **Julia Theil:** Writing – review & editing, Writing – original draft, Validation, Methodology. **Sybil Charrière:** Writing – review & editing, Validation, Methodology.

## Declaration of competing interest

None.

## Data Availability

Data will be made available on request.
